# Management of arterial injuries in endoscopic endonasal approaches

**DOI:** 10.3171/2020.4.FocusVid.19976

**Published:** 2020-04-01

**Authors:** Michael M. McDowell, Georgios Zenonos, Eric Wang, Carl H. Snyderman, Paul A. Gardner

**Affiliations:** Departments of 1 Neurological Surgery and; 2 Otolaryngology, University of Pittsburgh Medical Center, Pittsburgh, Pennsylvania

**Keywords:** vascular injury, endoscopic endonasal approach, trans-tuberculum, meningioma, video

## Abstract

This is the case of a 76-year-old woman presenting with progressive right vision loss consisting of a right eye temporal field cut and severe visual acuity loss. An MRI was performed showing a suprasellar mass for which she had been referred to our center for an endoscopic endonasal approach. The tumor was found to be densely adherent to adjacent structures, and an ophthalmic artery and A1–A2 junction injury were sustained during resection. The management of intraoperative vascular injuries is described.

The video can be found here: https://youtu.be/JJY6nYKTCSg.

## Transcript

**0:20** This is the case of a 76-year-old woman presenting with progressive right vision loss consisting of a right eye temporal field cut and severe visual acuity loss. An MRI was performed showing a suprasellar mass for which she had been referred to our center for endoscopic endonasal approach.

**0:40** On a contrasted T1 sequence we see a 2-cm homogenously enhancing lesion consistent with a planum sphenoidale meningioma. On CTA, the right A1 and bilateral proximal A2s are seen to be encased within the tumor.

**0:55** We begin with the resection of her right middle turbinate on the side of the endoscope. This is done in the usual fashion where it is initially transected approximately halfway down and then peeled down to its pedicle. Next, a left-sided vascularized nasoseptal flap was elevated. You can see here the inferior cut being made at the bottom of the choana, being extended down to the floor next to the nasal septum. Then the superior cut is made and extended superiorly anterior to the attachment of the middle turbinate to preserve olfactory fibers. Anteriorly, these are connected just posterior to the columella.

**1:37** The cut is made on a low-powered setting to avoid loss of olfaction or transection through the contralateral side. The flap is carefully elevated from the mucopericondrial plane. This is taken back to the sphenoid rostrum and then placed into the nasopharynx during the remainder of the case.

**2:01** A reverse flap is then prepared. The posterior septum is divided from the side of the nasoseptal flap. Care is taken to not perforate the contralateral mucosa, which is carefully raised posteriorly to the sphenoid before being transected and brought forward along the exposed left-sided cartilage.

**2:31** Two sutures are placed to secure the reverse flap before a wide sphenoidotomy is performed using a combination of Kerrison rongeurs to expose the sinus followed by the use of a high-speed drill with a 4-mm coarse diamond drill bit to shave down sphenoid septations and the sphenoid rostrum. This allows for good access to the tumor but also access to the paraclival ICA should proximal control become necessary.

**3:01** Once an adequately widened corridor has been made, the sellar face is carefully drilled and the bone removed. The planum sphenoidale is exposed by performing lateral osteotomies bilaterally from the medial opticocarotid recess up to the anterior planum osteotomy, which is determined based on individual tumor characteristics preoperatively and then confirmed with image guidance intraoperatively.

**3:29** Once thinned with an irrigating drill, the bone of the middle clinoid, parasellar carotids, and optic canals can be carefully elevated with a Kerrison rongeur and removed. Here is an anatomic dissection depicting the extradural exposure up to the level of the optic canals. Intradurally, the ophthalmic artery can be found to take off just distal the dural ring from the ICA.

**3:56** The origin of this artery can be highly variable and may arise superior or superomedially along the ICA as well as laterally. Generally, it is not advisable to make bites with the Kerrison.

**4:14** When a vascular injury has occurred, the first and most important step is to secure visualization with the endoscope by pulling it out of the stream of blood and using large suctions and irrigation to clear the field sufficiently to identify and compress the source of bleeding with a cottonoid. Prior to attempting to repair the injury, additional bone fragments are removed so as to not impede access to the source of bleeding. Bipolar electrocautery was then used to secure the avulsed artery. Once the major arterial bleeding is controlled, the field is cleared of blood to ensure appropriate orientation before proceeding.

**5:06** Surgifoam and additional use of bipolar are used to address residual minor bleeding.

**5:26** The prechiasmatic dura is opened sharply with curved scissors, exposing tumor immediately. Using a feather blade, the optic canal dura up to the medial falciform ligament is opened via transecting upward and laterally. Traveling in the direction reduces the risk of optic nerve injury. The tumor is centrally debulked and involved dura removed sequentially to allow for the eventual identification of the optic chiasm. Superior hypophyseal vessels are seen encased in tumor. The proximal left optic nerve is decompressed, but tumor remains adherent along the right optic nerve and the anterior cerebral arteries are not yet seen. The anterior margin of the tumor is then debulked and tumor is found to be very adherent to the invaded frontal lobe. The right olfactory tract is nicely visualized here.

**6:20** After further debulking, indocyanine green fluorescent endoscopy is used to identify the left A2, which is adherent to tumor. In order to follow the A2 back to the AComm and identify the right A2, tumor is carefully dissected off of the superior margin of the chiasm.

**6:54** Unfortunately, the right A1–A2 junction is not readily visualized. Here is an anatomic dissection depicting the relative relationship of the ACA vessels and the chiasm under normal circumstances. Tumor is dissected sharply off the anticipated location of the A1–A2 junction, but the wall is lacerated in the process.

**7:14** As before, visualization is secured and a suction is used to control the site of bleeding. Cottonoids are placed over exposed CSF spaces to minimize blood extravasation.

**7:29** A spot weld technique was planned but the vessel was poorly visualized, and so a clip was placed in attempt to secure the bleeding site. Unfortunately, the bulk of the tumor made clipping unsuccessful and a spot weld was performed using bipolar cautery.

**7:53** Additional tumor was debulked, but given the extent of tumor adherence it was decided that a cuff of tumor would be left on the proximal A2s to avoid further risk to the vasculature. The distal A2s were visualized and confirmed to be patent using fluoroscopy and Doppler.

**8:15** For closure a DuraGen inlay was tucked carefully within the dura as much as possible, but this was limited by tumor persistence. A fascia lata graft was harvested for additional layering over the defect, followed by a nasoseptal flap. Surgicel was applied along the nasoseptal flap margins and Gelfoam and Merocels were packed into the cavity.

**8:39** Postoperative MRI demonstrated expected tumor residual but good optic nerve decompression. Formal angiography demonstrated complete loss of the right ophthalmic artery. The right A2 was stenotic but patent. Postoperatively the patient’s vision remained stable but did not improve. After 24 hours of a mean arterial pressure floor of 85 and high-dose steroids she was ultimately transferred out of the ICU and eventually to rehab at her neurological baseline. Pathology was a WHO grade I meningioma. Her residual will be observed.

**9:18** Vascular injuries are a complication that is best approached via avoidance. Careful inspection of vessel takeoffs and trajectory in conjunction with thoughtful consideration of the goals of surgery in the context of patient age and quality of life are recommended when a high-risk lesion is surgically approached.

## Supplemental Information

Supplemental Figures
