# Supplementary material for: Management of arterial injuries in endoscopic endonasal approaches
Source: Neurosurg Focus Video. 2020 Apr 1;2(2):V4. doi: 10.3171/2020.4.FocusVid.19976 (PMC9542590; doi:10.3171/2020.4.FocusVid.19976)
Supplement: Supplemental Figures [file 19976.McDowell.NSVapr2020.supplmat.pdf]

Supplemental material

Management of arterial injuries in endoscopic endonasal approaches

Michael M. McDowell, MD,[1] Georgios Zenonos, MD,[1] Eric Wang, MD,[2] Carl H. Snyderman, MD, MBA,[2] and Paul A. Gardner, MD[1]

http://thejns.org/doi/abs/10.3171/2020.4.FocusVid.19976

DISCLAIMER *Neurosurgical Focus: Video* acknowledges that the following section is published verbatim as submitted by the authors and did not go through either the journal's peer-review or editing process.

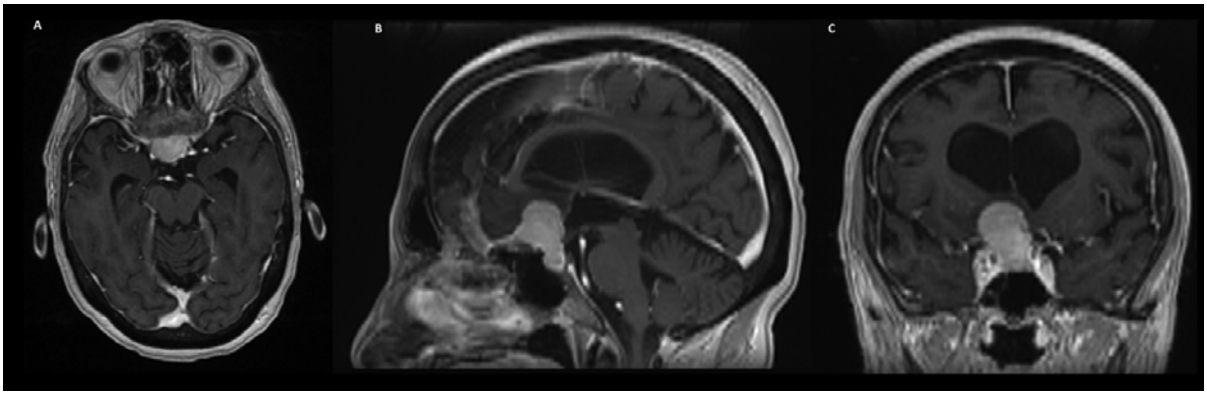

FIG. 1. T1-weighted MRI sequence with contrast in the axial (A), sagittal (B), and coronal (C) planes depicting a planum sphenoidale meningioma.

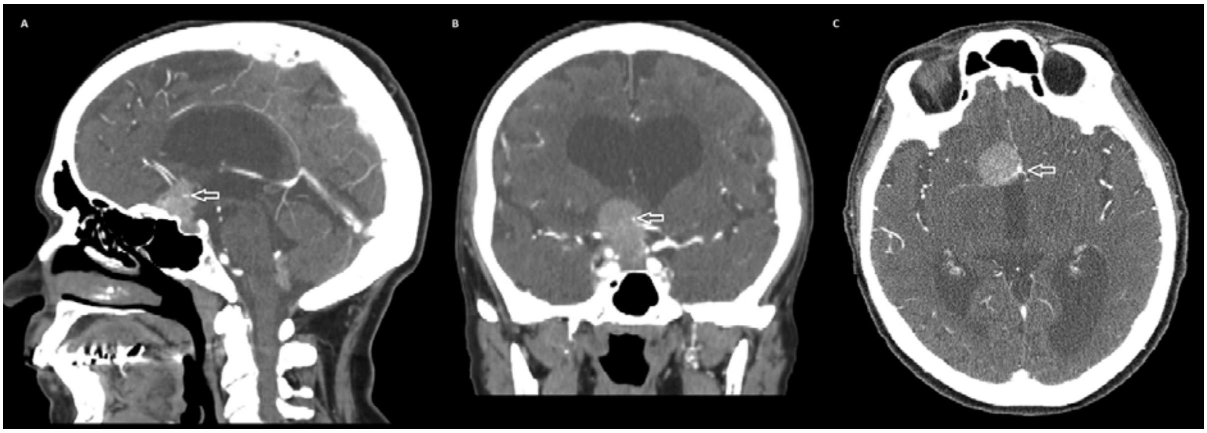

FIG. 2. CT angiogram in sagittal (A), coronal (B), and axial (C) planes depicting vascular encasement of the right and left anterior cerebral arteries by tumor.

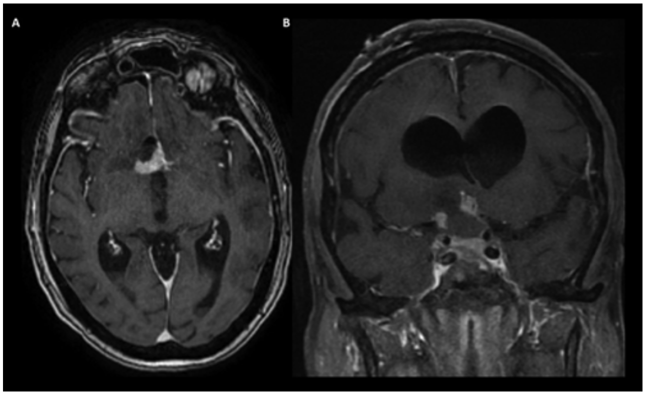

FIG. 3. T1-weighted MRI sequence with contrast in the axial (A) and coronal (B) planes depicting optic nerve decompression and expected tumor residual.
